# IL-10 Deficiency Aggravates Renal Inflammation, Fibrosis and Functional Failure in High-Fat Dieted Obese Mice

**DOI:** 10.1007/s13770-020-00328-7

**Published:** 2021-02-05

**Authors:** Dae Hwan Kim, So Young Chun, EunHye Lee, Bomi Kim, BoHyun Yoon, Haejung Gil, Man-Hoon Han, Yun-Sok Ha, Jun Nyung Lee, Tae Gyun Kwon, Bum Soo Kim, Byung Ik Jang

**Affiliations:** 1 Department of Laboratory Animal Research Support Team, Yeungnam University Medical Center, Daegu, 42415 Republic of Korea; 2 BioMedical Research Institute, Kyungpook National University Hospital, Daegu, 41944 Republic of Korea; 3 Department of Pathology, School of Medicine, Kyungpook National University, Daegu, 41944 Republic of Korea; 4 Department of Urology, Kyungpook National University Chilgok Hospital, Daegu, Korea; 5 Department of Urology, School of Medicine, Kyungpook National University, Daegu, Republic of Korea; 6 Department of Internal Medicine, School of Medicine, Yeungnam University, Daegu, Republic of Korea

**Keywords:** IL-10 deficiency, Renal failure, Obesity, High-fat diet

## Abstract

**BACKGROUND:**

High-fat diet-induced obesity is one of the major cause of chronic renal failure. This obesity-related renal failure is mainly caused by inflammatory processes. However, the role of the major anti-inflammatory cytokine interleukin (IL)-10 has not been researched intensively.

**METHODS:**

To evaluate the effect of IL-10 deficiency on obesity-related renal failure, the in vivo study was carried with four animal groups; (1) Low-fat dieted C57BL/6 mice, (2) Low-fat dieted IL-10 knockout (KO) mice, (3) High‐fat dieted C57BL/6 mice and (4) High‐fat dieted IL-10 KO mice group. The analysis was carried with blood/urine chemistry, H&E, Oil-Red-O, periodic acid-Schiff and Masson’s trichrome staining immunohistochemistry and real-time PCR methods.

**RESULTS:**

At week 12, high‐fat dieted IL-10 KO mice showed 1) severe lipid accumulation in kidneys, cholesterol elevation (in total, serum kidney) and low-density lipoprotein increasion through the SCAP-SREBP2-LDLr pathway; (2) serious histopathologic alterations showing glomerulosclerosis, tubulointerstitial fibrosis and immune cell infiltration; (3) increased pro‐inflammatory cytokines and chemokines expression; (4) enhanced renal fibrosis; and (5) serious functional failure with high serum creatinine and BUN and proteinuria excretion compared to other groups.

**CONCLUSION:**

IL-10 deficiency aggravates renal inflammation, fibrosis and functional failure in high-fat dieted obese mice, thus IL-10 therapy could be applied to obesity-related chronic renal failure.

## Introduction

High-fat dieted obesity is one of the major reason of chronic renal failure [1]. Infiltrated lipid into kidney causes renal pathogenic alterations and functional impairment. The main mechanism of obesity induced renal injury is inflammation [2]. In details, high-fat diet induces excessive visceral and ectopic lipids accumulation through the SCAP-SREBP2-LDLr pathway [3], the lipids are oxidized and caused cellular organelle disruption and macrophage activation. Infiltrated macrophages, injured renal cells and visceral/ectopic adipocytes secrete inflammatory cytokines, chemokines and adipokines, especially, c-reactive protein (CRP), interleukin (IL)-6, tumor necrosis factor (TNF)-α, leptin and serum amyloid A (SAA) [4]. These inflammatory molecules lead to glomerulosclerosis and tubulointerstitial fibrosis, which impair the glomerulus filtration and finally results chronic renal failure [2].

In obese conditions, anti-inflammatory mediators are significantly down-regulated. One of the most important anti-inflammatory cytokine is IL-10 [5]. IL-10 effectively inhibits pro-inflammatory cytokines and chemokines secreted by monocytes, macrophages or lymphocytes [6]. In normal kidneys, IL-10 reduces inflammation, mesangial cell proliferation, glomerulosclerosis and interstitial fibrosis [7–9]. In obese conditions, IL-10 synthesis is suppressed systemically and locally, resulting low-grade chronic inflammation [10]. Even though, IL-10 is known to be closely associated with obesity-related inflammation [7, 11], the effect of IL-10 deficiency in obesity-related renal failure has not been investigated intensively.

Therefore, we investigated the effect of IL-10 deficiency for renal injury through inflammation, fibrosis and functional analysis in high-fat dieted IL-10 knock-out (KO) mice. For this observation, lipid accumulation, lipid uptake pathway, immune cells infiltration, pro-inflammatory cytokines and chemokines secretion, fibrosis, histological alterations and functional failure in kidneys were analyzed. Understanding the effect of IL-10 deficiency in obesity could provide new therapeutic opportunity for renal.

## Materials and methods

### Animals and experimental protocol

This study followed the guidelines of Yeungnam University based on the Guide for the Care and Use of Laboratory Animals published by the US National Institutes of Health. The animal experiments were approved by the ethics committee of the Division of Laboratory Animal Science of Yeungnam University (YUMC-AEC2018-018).

IL-10-deficient (IL-10KO) male mice (5 weeks, C57BL/6 background) were purchased from Jackson Laboratory (Bar Harbor, ME, USA) and bred in the laboratory animal center at the yeungnam university school of Medicine. Mice were separated into four groups (n = 6 for each group). 1) Low/BL6: C57BL/6 mice fed the low-fat regular diet (D12450B, Research Diets, New Brunswick, NJ, USA); 2) Low/IL10KO: IL-10KO mice fed the low-fat diet; 3) High/BL6: C57BL/6 mice fed the high‐fat diet (with 60 kcal% fat, D12492, research diets); and 4) High/IL10KO: IL-10KO mice fed the high-fat diet. Body weight was measured weekly for 12 weeks and 24-h urine volume was measured one day before sacrifice with a metabolic cage (Tecniplast, Buguggiate, VA, Italy).

### Blood, urine and kidney chemistry

Before sacrifice, mice were fasted for 4 h, euthanized by CO_2_ inhalation and blood and urine were collected. Samples were centrifuged at 12,000 rpm at 4 °C for 10 min and supernatant was collected. The concentration of total cholesterol, serum cholesterol and low-density lipoprotein (LDL) were measured using an automated biochemical analyzer (Dimension RxL, Dade Behring, Inc, Deerfield, IL, USA). Measurements of proteinuria (Invitrogen, Carlsbad, CA, USA), blood urea nitrogen (BUN, Ortho-Clinical Diagnostics, Linden, NJ, USA), serum creatinine (Diazyme labs, Poway, CA, USA), CRP, IL-6, TNF-a, leptin (R&D systems, Minneapolis, MN, USA) and SAA (Invitrogen) were obtained using commercial kits according to the manufacturer’s instructions. For kidney cholesterol measurement, Cholesterol Assay Kit (Cell Biolabs, San Diego, CA, USA) was used. Total protein was extracted using a ReadyPrep™ protein extraction kit (Bio-Rad, Hercules, CA, USA) and protein concentration was analyzed using the Bradford method.

### Renal histology

After blood and urine collection, gross imaging of visceral fat was obtained. The kidneys were weighed and divided into two pieces for frozen and paraffin sectioning. With frozen Sections (10 μm with OCT compound), lipid accumulation was evaluated with an Oil Red O staining kit (Abcam, Cambridge, UK) following the manufacturer’s instructions and staining quantification was carried with a spectrophotometer at 492 nm absorbance. With paraffin Sections (5 μm), H&E, periodic acid-Schiff (PAS) and Masson’s trichrome staining were conducted. H&E staining was performed using routine methods and PAS (Abcam) and Masson’s trichrome staining (Sigma-Aldrich, St. Louis, MO, USA) were performed using commercial kits following the manufacturer’s instructions. Glomerulus size and number, mesangial cell expansion, glomerulosclerosis and tubulointerstitial fibrosis were determined by an experienced pathologist.

### Immunohistochemistry (IHC)

Immunohistochemical studies were performed to detect F4/80, CD3, α-smooth muscle actin (SMA), collagen type 1 (Col1) and fibronectin. Endogenous peroxidases were inactivated using 3% H_2_O_2_, followed by blocking with goat serum. Sections were incubated overnight at 4 °C with primary antibody (1:200, Abcam), incubated for 45 min with secondary antibody (Alexa Fluor 594, Life Technology, Waltham, MA, USA) and DAPI was used for nuclei staining. The target protein was quantified using the image pro plus software at × 400 magnification from 6 renal sections per group.

### Quantitative real-time PCR

Half of the kidney tissue was homogenized in TRIzol reagent and total RNA was extracted according to the TRIzol protocol (Takara Life Technologies, Shiga, Japan). Total RNA (2 μg) was subjected to DNase digestion followed by cDNA synthesis using a kit (Bio-Rad). Real-time PCR was performed using SYBR Green PCR master mix (Bio-Rad). GAPDH served as an internal standard for data normalization. The amount of mRNA was calculated using the comparative C_T_ method. The PCR reactions were performed followed using 40 PCR cycles, with each cycle at 95 °C for 15 s, 60 °C for 1 min and 72 °C for 1 min. Primers were designed by primer express software V2.0 (Applied biosystems, Foster City, CA, USA) and were listed in Table 1 along with gene function.

**Table 1:** Primer sequences for real-time PCR

Gene	Function	Sequences
LDLr (Low-density lipoprotein receptor)	Cholesterol uptake receptor	S: 5′-CATCCTCGGACATCCACCC-3′ AS: 5′-TTCGGTCGTGGCACAAGAAC-3′
SREBP (sterol regulatory element-binding protein-2)	LDLr regulator in mesangial cells	S: 5′-GTTGACCACGCTGAAGACAGA-3′ AS: 5′-CACCAGGGTTGGCACTTGAA-3′
SCAP (SREBP-cleavage activating protein)	A chaperone of SREBP	S: 5′-CAGTATGTGTTGCTCCACAAAA-3′ AS: 5′-AGGGTCCGTCATCTCAGTCAC-3′
F4/80	Macrophage marker	S: 5′-AGTCCTGAGTTGCACGTACA-3′ AS: 5′-CAACCTGCCACAACACTCTC-3′
CD3	T cell marker	S: 5′-TAGGCACCATATCCGGCTTTA-3′ AS: 5′-CATCCTGTCCCGCAATGAGA-3′
CRP (C-reactive protein)	Pro-inflammatory factors	S: 5′-TGTGTTGGAGCCTCAGGAAT-3′ AS: 5′-CGCAGCTTCAGTGTCTTCTC-3′
IL-6		S: 5′-AGTTGCCTTGGGACTGA-3′ AS: 5′-TCCACGATTTCCCAGAGAAC-3′
TNF-α (tumor necrosis factor-alpha)		S: 5′-TTCACTGGAGCCTCGAATGT-3′ AS:5′-ACCTGACCACTCTCCCTTTG-3′
Leptin		S: 5′-AGATCTACACCAGGGACCCT-3′ AS: 5′-GCCCCACATTTGAGACAGTG-3′
α-SMA (alpha-smooth muscle actin)	Fibrosis indicator	S: 5′-CTGACAGAGGCACCACTGAA-3′ AS: 5′-CATCTCCAGAGTCCAGCACA-3′
Col1 (collagen type 1)		S: 5′-GAGCGGAGAGTACTGGATCG-3′ AS: 5′-GCTTCTTTTCCTTGGGGTTC-3′
Fibronectin		S: 5′-AATGGAAAAGGGGAA-3′ AS: 5′-CTCGGTTGTCCTTCTTGCTC-3′
GAPDH	Housekeeping gene	S: 5′-TGTGTCCGTCGTGGATCTGA-3′ AS: 5′-CCTGCTTCACCACCTTCTTGA-3′

### Data analysis

Data were evaluated for statistical significance using two-sided paired *t*-tests by SPSS 13.0 Software (IBM, New York, NY, USA). Values were present as means ± SD and a difference was considered significant if the *p* value was < 0.05.

## Results

### Lipid accumulation

**Fig. 1:**
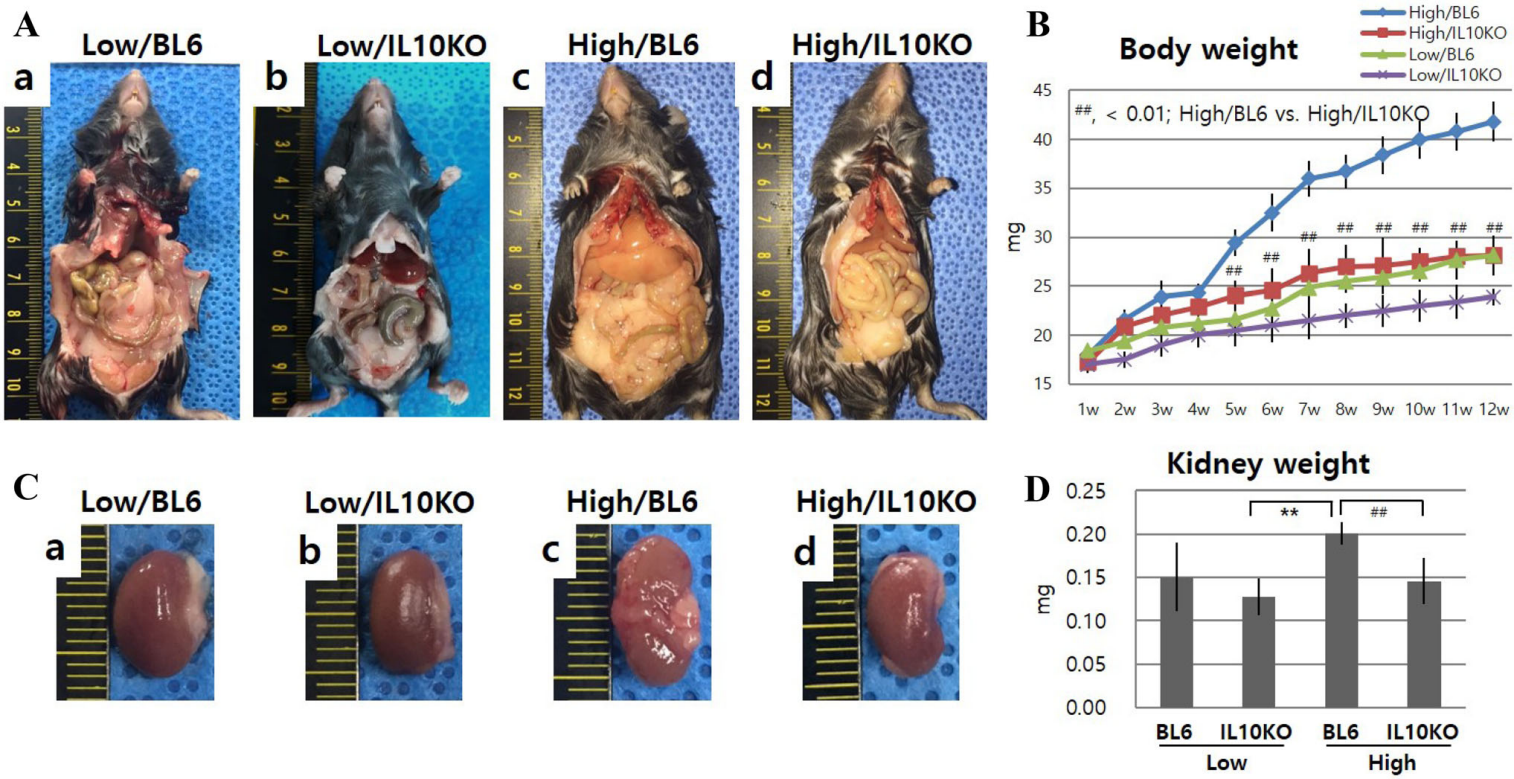
Influence of IL-10 deficiency and high-fat diet on lipid accumulation in the body and kidneys. **A** Representative gross images of visceral fat at week 12, **B** Body weight over 12 weeks, **C** Representative gross images of the kidney at week 12, **D** Kidney weight at week 12. High-fat diet leads to abundant visceral fat accumulation compared to low-fat diet. IL-10 genetic KO causes reduced fat accumulation. Low/BL6; C57BL/6 mice fed a low-fat diet, Low/IL10KO; IL-10KO mice fed a low-fat diet, High/BL6; C57BL/6 mice fed a high‐fat diet, High/IL10KO; IL-10KO mice fed a high-fat diet. Data are expressed as means ± SD and analyzed by Student’s *t*-tests (*n* = 6 per group). **, *p* < 0.01 versus Low/IL10KO; ^##^, *p* < 0.01 versus High/BL6

**Fig. 2:**
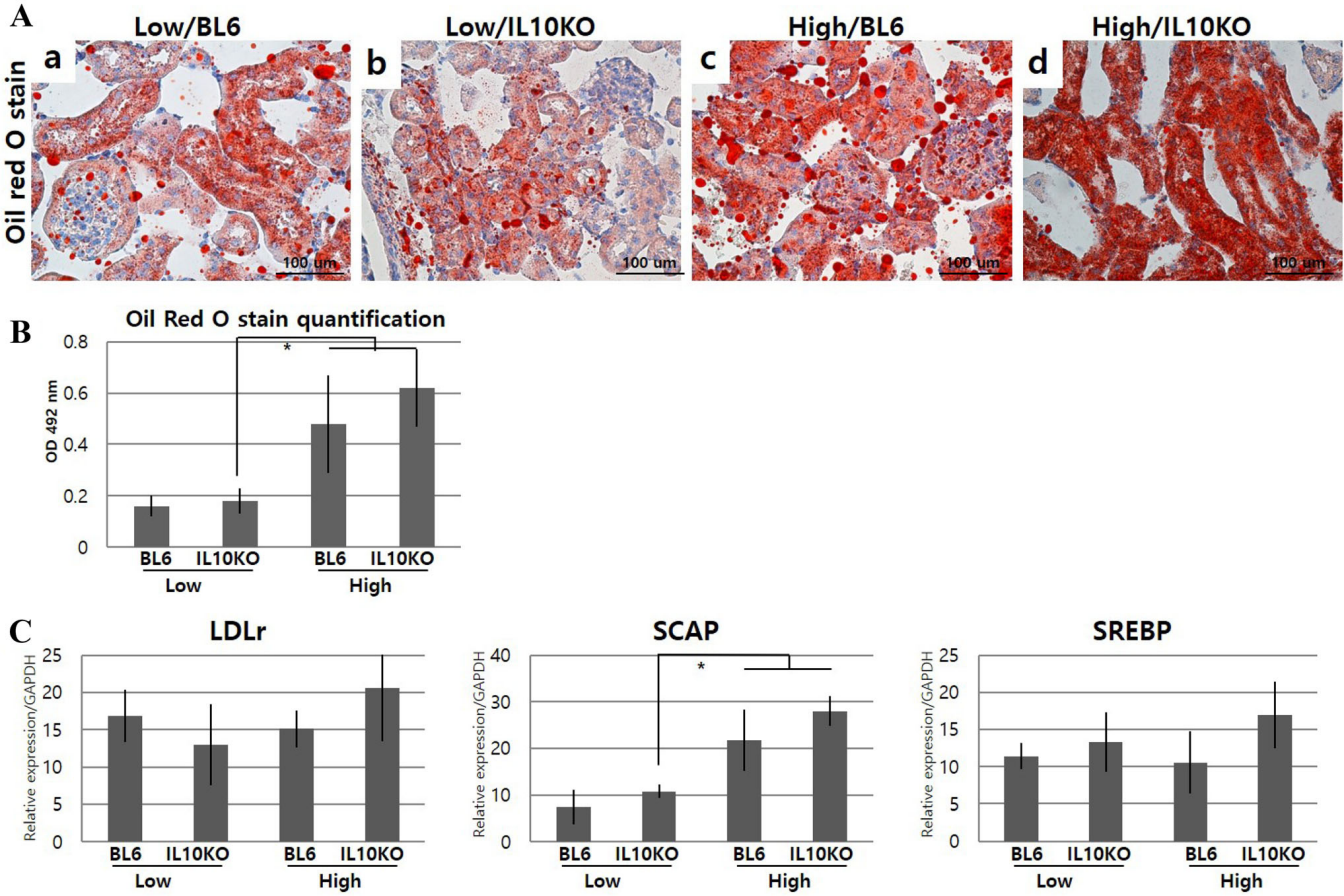
Influence of IL-10 deficiency and high-fat diet on renal lipid accumulation pathways. **A** Oil droplets in kidneys as detected by Oil red O staining, **B** Quantification of Oil red O staining, **C** SCAP-SREBP2-LDLr gene expression for evaluation of lipid accumulation pathways. IL-10 deficiency and high-fat diet aggravate renal lipid accumulation and increased the expression of SCAP-SREBP2-LDLr pathway-related genes, which were identified by Oil red O staining and real-time PCR, respectively. Low/BL6; C57BL/6 mice fed a low-fat diet, Low/IL10KO; IL-10-KO mice fed a low-fat diet, High/BL6; C57BL/6 mice fed a high‐fat diet, High/IL10KO; IL-10-KO mice fed a high-fat diet. Data are expressed as mean ± SD and analyzed by Student’s *t*-tests (*n* = 6 per group). *, *p* < 0.05 versus Low/IL10KO. All fields were chosen from the cortex and outer medulla

High-fat diet leads to fat accumulation in multiple internal organs and under the skin, whereas IL10-KO mice showed reduced fat mass compared to the High/BL6 mice (Fig. 1A c, d). High/BL6 mice showed a steady increase in body weight over the 12 week observation period, while High/IL10KO mice showed significantly less weight gain compared to High/BL6 mice from week 5 (*p* < 0.01, Fig. 1B). In the gross images of kidneys, high-fat dieted mice showed a pale color compared to low-fat diet mice and IL10-KO mice showed smaller perirenal fat pads, reduced kidney size (Fig. 1C, D) and lower weight (Fig. 1D, *p* < 0.01) than High/BL6 at week 12. In the Oil Red O staining, High/IL10KO mice showed enhanced lipid droplets in the kidney compared to High/BL6 (Fig. 2). In the Oil Red O stain quantification, high-fat dieted mice showed a significantly increased extract concentration compared to low-fat dieted mice (*p* < 0.05, Fig. 2B). In the quantification of systemic lipid levels by total cholesterol, serum cholesterol and LDL values, High/IL10KO mice showed significantly increased concentrations of all lipids compared to High/BL6 (*p* < 0.01, Table 2). In the analysis of lipid uptake pathways, mRNA expression levels of SCAP, SREBP2 and LDLr in High/IL10KO kidneys were relatively increased values compared to the High/BL6 mice (Fig. 2C).

**Table 2:** Influence of IL-10 deficiency and high-fat diet on systemic and local lipid concentrations

ELISA	Low		High	
	BL6	IL10KO	BL6	IL10KO
Total cholesterol (mmol/L)	1.17 ± 0.40	3.86 ± 0.20	47.34 ± 3.82**	86.32 ± 5.60**^,##^
Serum cholesterol (mg/dL)	127.85 ± 15.82	97.47 ± 25.31	156.96 ± 6.33**	222.78 ± 16.45**^,##^
Low-density lipoprotein (pg/mL)	6.97 ± 0.1	7.23 ± 0.2	72.79 ± 5.22**	98.52 ± 14.29**^,##^
Kidney cholesterol (μg/mg protein)	10.55 ± 1.32	17.7 ± 5.2	22.5 ± 2.60	22.92 ± 5.14

Low; low-fat diet, High; high‐fat diet with 60 kcal% fat, BL6; C57BL/6 mice, IL10KO; IL-10KO mice (each group, *n* = 6). Values are means ± SD. **, *p* < 0.01 versus Low/IL10KO; ^##^, *p* < 0.01 versus High/BL6

### Renal histopathological alterations

**Fig. 3:**
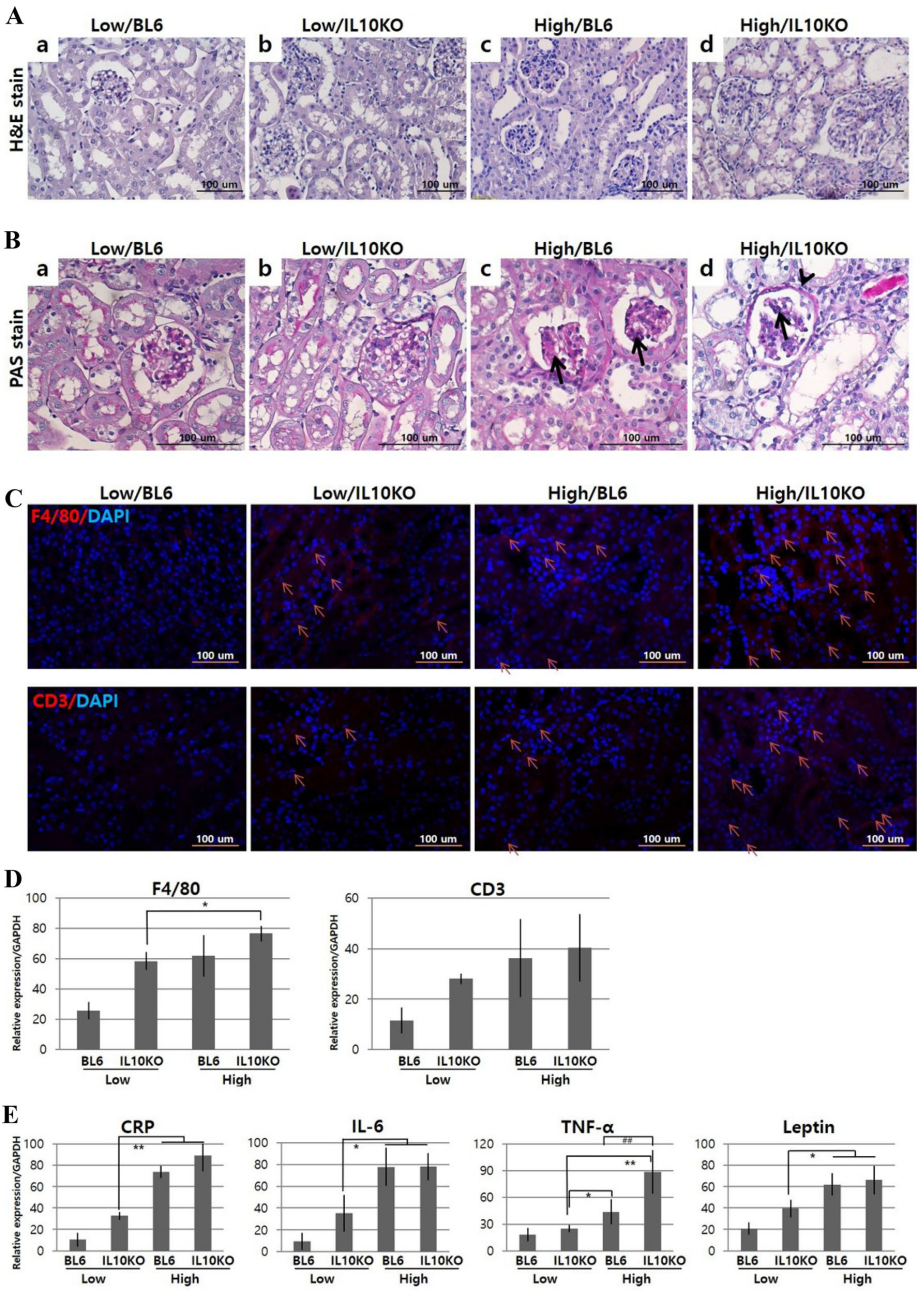
Influence of IL-10 deficiency and high-fat diet on renal inflammatory responses. **A** H&E staining for histopathological alterations and immune cell infiltration, **B** PAS staining for glomerulus sclerosis (arrow) and basement membrane thickness (arrowhead); **C** Immunohistochemistry for the identification of F4/80 + macrophages and CD3 + T-cells; **D** Quantitative real-time PCR for F4/80 and CD3 genes; **E** Quantitative real-time PCR for pro-inflammatory mediators. IL-10 deficiency and high-fat diet aggravates renal inflammatory responses with immune cell infiltration and pro-inflammatory molecule secretion, which were identified by increases in inflammation-related markers, F4/80, CD3, CRP, IL-6, TNF-α, MCP-1, ICAM-1 and RANTES, through IHC and real-time PCR analysis. Low/BL6: C57BL/6 mice fed a low-fat diet; Low/IL10KO: IL-10KO mice fed a low-fat diet; High/BL6; C57BL/6 mice fed a high‐fat diet; High/IL10KO; IL-10KO mice fed a high-fat diet. Data are expressed as mean ± SD and analyzed by Student’s *t*-tests (*n* = 6 per group). *, *p* < 0.05 versus Low/IL10KO; **, *p* < 0.01 versus Low/IL10KO; ^##^, *p* < 0.01 versus High/BL6. All fields were chosen from the cortex and outer medulla

To evaluate the effects of IL-10 deficiency on renal histological alterations, H&E- (Fig. 3A) and PAS-(Fig. 3B) stained kidney sections were examined. When compared to the control mice, high-fat dieted and/or IL-10-deficient mice showed obvious histological changes at glomerulus and tubules, such as glomerulomegaly, glomerulosclerosis (arrow, Fig. 3B, C, D), glomerular capillary dilation, thickened glomerular basement membrane (arrow head, Fig. 3B, D), mesangial matrix expansion, podocyte hypertrophy, tubule lumens enlargement, interstitial cell necrosis and shortened tubular epithelium. High/IL10KO kidneys showed more severe renal obstruction with a fibrotic cortex, sclerotic glomeruli, necrotic renal papilla, thickened and narrowed arteries, dilated tubules with pink casts, ragged epithelium undergoing necrosis and degenerated vacuolar structures.

To quantify histological alterations, glomerular area and numbers were measured (Table 3). High/IL10KO mice showed the largest glomerular area, followed by High/BL6, Low/IL10KO and Low/BL6. When count of glomerulus number, all groups showed similar values. Using PAS staining, sclerotic glomeruli were indexed as per total glomerulus (%) (Fig. 3B, Table 3) and the sclerotic lesions were indicated with arrows. In High/IL10KO mice, 17.17% of the glomeruli were segmentally sclerosed, High/BL6 was 14.93%, Low/BL6 was 1.16% and Low/IL10KO was 2.04%. The number of mesangial cells in glomeruli was highest in High/IL10KO mice and followed High/BL6, Low/IL10KO and Low/BL6 group.

**Table 3:** Influence of IL-10 deficiency and high-fat diet on renal histopathologic alterations

Histopathology	Low		High	
	BL6	IL10KO	BL6	IL10KO
Glomeruli area (μm^2^)	8391.79 ± 4119.8	8979.84 ± 2933.6	10,182.6 ± 4596.5	11,586.6 ± 3158.3
Glomerulus no	91.67 ± 6.25	92.00 ± 7.81	93.33 ± 13.72	97.33 ± 5.92
Sclerotic glomerulus (%)	1.16 ± 0.31	2.04 ± 0.11	14.93 ± 5.61**	17.17 ± 7.22
Mesangial cell no/glomerulus	8.54 ± 2.23	10.63 ± 3.22	13.35 ± 3.77	14.36 ± 3.60
Interstitial fibrosis (%)	0.51 ± 0.01	1.52 ± 0.3	8.17 ± 2.5**	9.25 ± 1.7**

Low: low-fat diet, High: high‐fat diet with 60 kcal% fat, BL6: C57BL/6 mice, IL10KO: IL-10KO mice (each group, *n* = 6). Values are means ± SD. **, *p* < 0.01 versus Low/IL10KO

### Inflammatory cell infiltration

In H&E staining, Low/BL6 kidneys did not show any immune cell infiltration (Fig. 3A a). However, High/IL10KO mice (Fig. 3A d) showed that abundant polymorphonuclear neutrophil leukocytes in renal tubules and some leukocytes formed a cast within the tubule. The accumulation of pale macrophages break down the renal parenchyma with ongoing inflammation. Macrophage infiltration was prominent in glomeruli. Eosinophils, neutrophils and mononuclear cells were scattered in the interstitium and lymphocytes were observed surrounding the vessels. Macrophages and T cells infiltration (identified with F4/80 and CD3 antibody in IHC) were significantly observed in the High/IL10KO (Fig. 3C). The F4/80 positive cell number per each group was 5 ± 1.25, 25 ± 0.97, 31 ± 3.693 and 41 ± 2.67 for Low/BL6, Low/IL10KO, High/BL6 and High/IL10KO, respectively. The CD3 positive cell number per each group was 6 ± 1.21,12 ± 2.14, 11 ± 1.39 and 24 ± 1.81 for Low/BL6, Low/IL10KO, High/BL6 and High/IL10KO, respectively. F4/80 and CD3 gene expression with real-time PCR also showed increased expression in High/IL10KO mice compared to other groups (Fig. 3D).

### Pro-inflammatory cytokines and chemokines

To evaluate the inflammatory cytokines and chemokines (secreted by infiltrating immune cells, ectopic adipocytes and/or resident renal cells), their serum concentrations (systemic) and renal mRNA expression (local) were analyzed. High/IL10KO mice showed comparatively increased serum levels of IL-6, TNF-α, leptin and SAA (Table 4). Renal mRNA levels of pro-inflammatory molecules were significantly elevated in high-fat dieted mice and more increased in the IL-10 deficient condition (Fig. 3E), especially, High/IL10KO mice showed a significantly higher expression of TNF-α than High/BL6 mice (*p* < 0.01).

**Table 4:** Influence of IL-10 deficiency and high-fat diet on the concentrations of pro-inflammatory mediators in serum

ELISA	Low		High	
	BL6	IL10KO	BL6	IL10KO
CRP (pg/mL)	30.071 ± 7.41	109.69 ± 2.17	175.84 ± 8.85**	176.61 ± 15.35**
IL-6 (pg/mL)	30.99 ± 16.46	44.37 ± 10.40	50.61 ± 13.77	54.31 ± 8.87*
TNFα (pg/mL)	99.10 ± 5.77	123.22 ± 16.22	126.6 ± 16.18	141.94 ± 15.90**
Leptin (μg/mL)	9.09 ± 7.00	13.92 ± 0.23	18.68 ± 5.07**	18.70 ± 1.86**
SAA (ng/mL)	290.17 ± 40.23	307.06 ± 51.89	434.43 ± 51.62**	539.36 ± 77.09**^,#^

Low: low-fat diet, High: high‐fat diet with 60 kcal% fat, BL6: C57BL/6 mice, IL10KO: IL-10-KO mice (each group, *n* = 6). Values are means ± SD. *, *p* < 0.05 versus Low/IL10KO; **, *p* < 0.01 versus Low/IL10KO; ^#^, *p* < 0.05 versus High/BL6

### Renal fibrosis

**Fig. 4:**
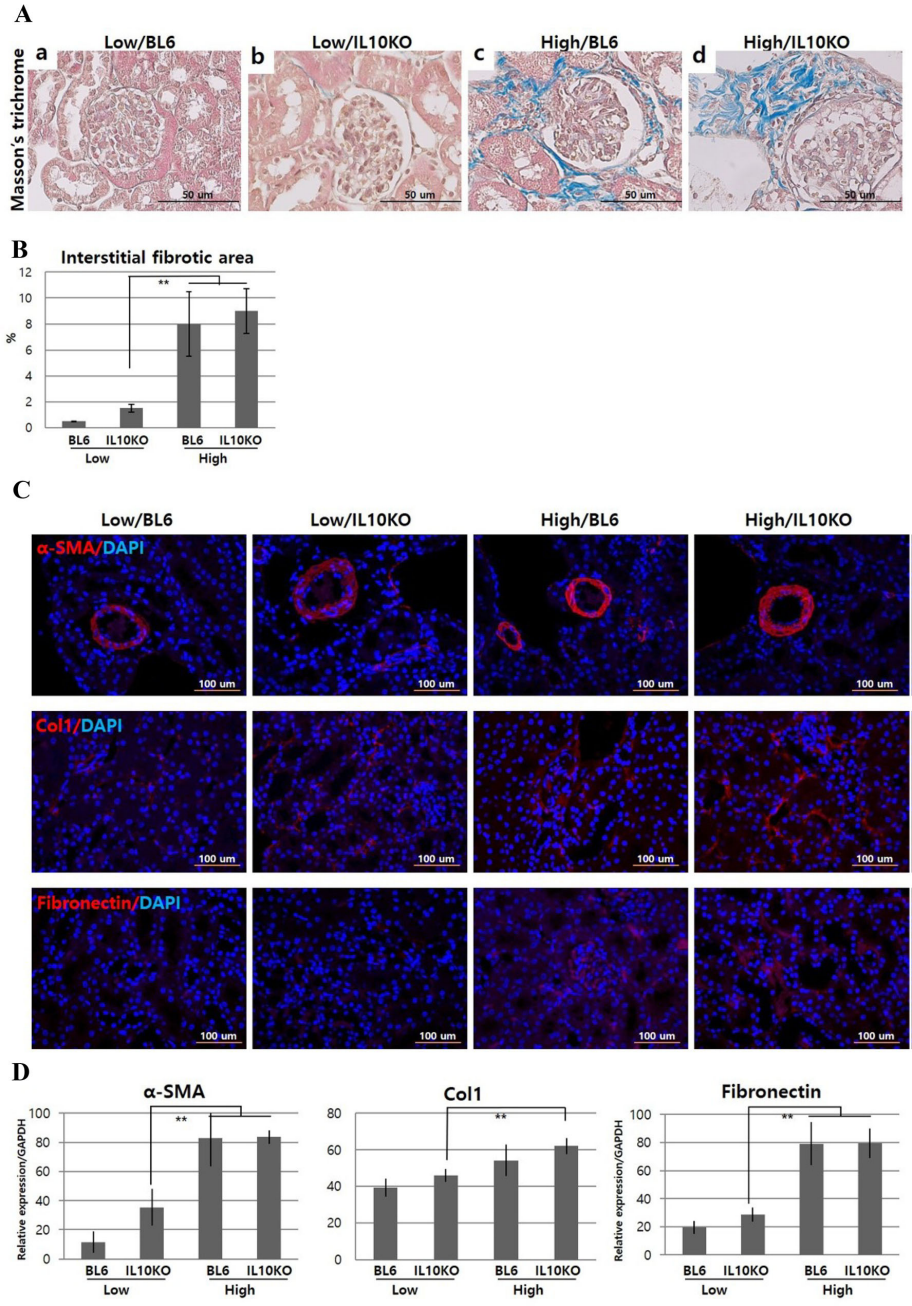
Influence of IL-10 deficiency and high-fat diet on renal fibrosis. **A** Masson’s trichrome staining for fibrosis in the glomerular and interstitial tubular area; **B** Percentage of interstitial fibrotic area; **C** Immunohistochemistry for the identification of deposited matrix; **D** Quantitative real-time PCR for fibrosis related genes. α-SMA: alpha smooth muscle actin, Col1: collagen type 1. IL-10 deficiency and high-fat diet aggravates tubulointerstitial ECM deposition as identified with Masson’s trichrome staining and the fibrosis related markers α-SMA, Col1 and fibronectin evaluated through IHC and real-time PCR analysis. Low/BL6: C57BL/6 mice fed a low-fat diet, Low/IL10KO: IL-10KO mice fed a low-fat diet, High/BL6: C57BL/6 mice fed a high‐fat diet, High/IL10KO: IL-10KO mice fed a high-fat diet. Data are expressed as the mean ± SD and analyzed by Student’s *t*-tests (*n* = 6 per group). *p < 0.05 versus Low/IL10KO; **p < 0.01 versus Low/IL10KO. All fields were chosen from the cortex and outer medulla

Inflammatory reactions lead to fibrosis and the lesion is mainly the cortical area (at the edge of the cortex and medullary ray). High/IL10KO mice showed expanded and severe fibrosis (Fig. 4A) in the interstitial area with Masson’s trichrome staining. The glomerular and interstitial fibrosis area was greater in High/IL10KO mice compared to the High/BL6 group (Fig. 4B, Table 3). In IHC, α-SMA was detected in vascular smooth muscle cells in arteries and arterioles and Col1 and fibronectin were usually found in the renal tubulointerstitium; the positive area was the highest in the High/IL10KO mice (Fig. 4C). The mRNA expression levels of α-SMA, Col1 and fibronectin were also significantly elevated in the high-fat dieted mice and more increased in the IL-10 deficient condition (*p* < 0.01, Fig. 4D).

### Renal function

Histopathologic alterations, inflammatory cytokines/chemokines secretion and prominent fibrosis are closely related to kidney functional abnormalities. Representative renal function indicators are serum creatinine, BUN and proteinuria, thus, we evaluated these factors with ELISA kits (Table 5). Serum creatinine was significantly higher in the high-fat dieted mice compared to low-fat dieted mice and IL-10 deficiency caused severely increased serum creatinine levels (*p* < 0.01). BUN and proteinuria values were not significant, but relatively elevated in high-fat dieted and IL-10 deficient mice.

**Table 5:** Influence of IL-10 deficiency and high-fat diet on renal function

ELISA	Low		High	
	BL6	IL10KO	BL6	IL10KO
Serum creatinine (ng/mL)	30.63 ± 6.04	38.12 ± 4.26	77.03 ± 8.39**	92.60 ± 2.56**,^##^
Blood urea nitrogen (mg/dL)	13.98 ± 4.13	17.38 ± 5.03	18.57 ± 4.16	21.59 ± 4.54*
Proteinuria (ng/mL)	1.7 ± 0.6	2.4 ± 0.5	3.4 ± 0.3**	3.6 ± 0.6**

Low: low-fat diet, High: high‐fat diet with 60 kcal% fat, BL6: C57BL/6 mice, IL10KO: IL-10-KO mice (each group,* n* = 6). Values are means ± SD, *p < 0.05 versus Low/IL10KO; **p < 0.01 versus Low/IL10KO; ^##^*p* < 0.01 versus High/BL6

## Discussion

We proved that IL-10 deficiency aggravates renal failure in obese condition through lipid accumulation, renal histopathology, inflammatory cells infiltration, pro-inflammatory cytokines/chemokines secretion, fibrosis and functional analysis.

When observing the fat mass in the body cavity, mice on a high-fat diet had more accumulation than mice on a low-fat diet, of course. However, contrary to expectations, the fat accumulation of IL-10 deficient mice was smaller than that of mice carrying this gene. It seemed as if IL-10 deficiency reduced fat accumulation. Unlike this, High/IL10KO mice showed significantly elevated serum cholesterol, renal lipid droplets and lipid uptake related genes expression. To interpret these unexpected results, we paid attention to the correlation between fat accumulation, inflammatory response and IL-10 deficiency. Inflammatory responses require a huge energy consumption, which is generated from adipose tissue degradation [12]. Also, the lipid accumulation at ectopic sites produces harmful lipid intermediates and these toxic metabolites cause severe lipotoxicity [13, 14]. The lipotoxic environment alters the immune phenotype of adipose tissue, leading to macrophage infiltration, pro-inflammatory cytokine secretion and apoptotic death of adipocytes [15]. Thus, the decreased fat mass seen in High/IL10KO mice may actually indicate a severe inflammatory response, due to IL-10 deficiency. In addition, the kidneys showed a severe inflammatory reaction. The High/IL10KO group showed reduced kidney size with atrophy, which indicates renal fibrosis following inflammatory reactions. Therefore, to confirm the severe inflammatory response by IL-10 deficiency, we performed histopathology, immune cell infiltration, pro-inflammatory cytokines/chemokines secretion and renal function analysis.

In the histopathologic analysis, high-fat diet showed severe mesangium hyperplasia and glomerulosclerosis compared to the low-fat diet. These histologic abnormalities were closely related to lipid levels in the serum and kidney. Increased serum LDL level stimulates the low-density lipoprotein receptor (LDLr) to take up cholesterol into kidney. That activity is regulated by sterol regulatory element binding protein-2 (SREBP-2) in mesangial cells and SREBP-cleavage activating protein (SCAP) is required as a chaperone protein [16, 17]. The enhanced lipid deposition in the kidney stimulates inflammatory cytokine secretion by renal mesangial cells [18, 19], promotes glomerulosclerosis by macrophages [20], adhesive factors produce by endothelial cells and chemokines produce by monocytes [21]; thus, excessive serum lipid levels cause mesangium proliferation, matrix synthesis, glomerular area increase and glomerular basement membrane thickening [22, 23]. The increased droplets in kidneys indicate the production of pro-inflammatory adipokines by ectopic adipocytes, which lead to monocyte recruitment and consequent glomerulosclerosis [2]. These histological alterations caused by high-fat diet were worsened under IL-10 deficient condition. Thus, above results demonstrated that IL-10 deficiency closely related to severe renal histopathologic alterations.

In the analysis of immune cell infiltration, high-fat diet and IL-10-deficient condition showed severely accumulation of F4/80^+^ macrophages and CD3^+^ T-cells in the interstitial areas of the cortex. Obesity by high-fat diet enhances the recruitment of macrophages to adipocytes and these macrophages secrete pro-inflammatory molecules [2, 24]. Considering IL-10 is an effective anti-inflammatory mediator against macrophages [25, 26], IL-10 deficiency leads to greater immune cell infiltration resulting in severe renal inflammatory responses.

The infiltrated immune cells release pro-inflammatory cytokines and chemokines. Ectopically accumulated adipocytes also release pro-inflammatory adipokines systemically and locally [27]. As well, injured renal cells secrete a wide range of inflammatory mediators [28–32]. Among the secreted pro-inflammatory molecules, CRP, IL-6, TNF-α, leptin and SAA are closely related to renal injury [33, 34]. These inflammatory molecules affect to the adhesion molecules and then stimulate neutrophils, macrophages, T cells, NK cells and dendritic cells, which further exacerbate the inflammation [28, 30, 31, 35–37]. In these cytokines and chemokines analysis, IL-10 deficient condition further increased these expressions.

These inflammatory molecules also stimulate fibroblast proliferation and extracellular matrix (ECM) production [38]. The deposited ECM types are fibrillar collagens (collagens I, III), glycoproteins (fibronectin, fibrillin, elastin, proteoglycans) [38] and nonfibrillar collagen (collagen IV) [39, 40]. Excessive accumulation of ECM reconstructs parenchymal tissue into connective tissue, which interrupts the original renal architecture and causes functional abnormalities. These fibrosis and ECM accumulation were also significantly increased under the condition of IL-10 deficiency.

Finally, the influence of IL-10 deficiency in obesity was analyzed for renal function. Creatinine and BUN are representative marker of renal functionality [41]. These are nitrogenous end products of metabolism and are removed from the blood by glomerular filtration and proximal tubular secretion, so, can reflect the glomerular filtration rate [41]. Another renal function marker is proteinuria (total protein in the urine) that indicates the filtration ability of the glomerulus [42, 43]. The high-fat dieted mice showed increased serum creatinine, BUN and proteinuria, which means renal endothelial cell dysfunction by the glomerulosclerosis and tubulointerstitial fibrosis and IL-10 deficiency worsens them.

The present study demonstrates that IL-10 deficiency aggravates inflammatory response, pro‐inflammatory/fibrosis cytokines/chemokines activation, immune cell infiltration, glomerulosclerosis, tubulointerstitial fibrosis, serum cholesterol, LDL, creatinine, BUN and proteinuria excretion in high-fat diet condition. Therefore, IL-10 treatment could be a potential therapy to obesity-related renal failure.
